# Pedagogy of Empowerment; Critical Learning Approaches in Community Health Promotion: An Umbrella Review

**DOI:** 10.34172/hpp.48231

**Published:** 2026-08-21

**Authors:** Narkabilova Gulnoza, Jabbarov Ulugbek, Tangirov Eldor, Rakhmatova Dildora, Shermukhamedov Rakhmatulla, Zubaydillo Narziyev, Nafosat Urokova, Nodirbek Komilov

**Affiliations:** ^1^Professor of Primary Education Methodology at Fergana State University,Uzbekistan; ^2^Associate Professor Scientific Research Institute of Family and Gender, Tashkent, Uzbekistan; ^3^Department of Pedagogy and Psychology, Samarkand State Medical University, Samarkand, Uzbekistan; ^4^Department of Social Sciences the Zarmed University, Bukhara, Uzbekistan; ^5^Department of Technological Education, Chirchik State Pedagogical University; ^6^Department of Social Sciences, Bukhara State Pedagogical Institute, Bukhara, Uzbekistan; ^7^Department of Uzbek Language and Literature, Bukhara State University, M. Iqbol, 11, 200100, Bukhara, Uzbekistan; ^8^Department of Quality Assurance, Fergana Medical Institute of Public Health, Fergana, Uzbekistan

**Keywords:** Empowerment, Health promotion, Critical pedagogy, Community-based participatory research, Health literacy

## Abstract

**Introduction:**

Critical, empowerment-based pedagogical approaches are widely advocated in health promotion, yet the evidence base remains fragmented across disciplines. This umbrella review systematically synthesizes existing systematic review evidence to clarify how these approaches are conceptualized, implemented, and evaluated across community health promotion contexts.

**Methods:**

A systematic search of five electronic databases (PubMed/MEDLINE, CINAHL, Scopus, ERIC, Web of Science) identified reviews published between January 2016 and December 2025. Eligible studies included systematic and scoping reviews examining critical pedagogy, empowerment education, critical health literacy, participatory action research, or transformative learning in health promotion. Methodological quality was assessed using AMSTAR-2. Findings were synthesized narratively.

**Results:**

Fifteen reviews (representing over 480 primary studies) met inclusion criteria; 80% were rated high quality. Theoretical frameworks spanned Freirean critical pedagogy (explicit in 53% of reviews), Youth Participatory Action Research, Community-Based Participatory Research, and transformative learning. Pedagogical tools varied by setting: community-based and youth-focused reviews employed visual methods (Photovoice, visual mapping), culturally grounded conversational frameworks (Talanoa), and dialogical approaches; educational interventions utilized problem-posing education, critical reflection, and student-led projects; clinical interventions employed structured psychoeducational modules and counseling. Interventions demonstrated consistent individual-level gains (self-efficacy, agency, critical thinking). Community-based participatory approaches additionally generated collective outcomes (social cohesion, community advocacy) and structural changes (organizational safety improvements, gender norm transformation). Clinical interventions showed individual empowerment but limited collective impact. Measurement of community- and organizational-level outcomes remained underdeveloped.

**Conclusion:**

Empowerment-based interventions grounded in critical pedagogy yield multidimensional benefits across individual, collective, and structural domains. Realizing full transformative potential requires addressing measurement gaps, ensuring cultural adaptation, and moving beyond individual-level outcomes. Integration of participatory frameworks across health, education, and workplace settings is warranted.

## Introduction

 Health promotion has long been characterized by a persistent conceptual divide concerning the purpose of educational intervention: whether such intervention should primarily transmit health-related information to recipients positioned as passive, or whether it should instead cultivate the critical capacities that enable individuals and communities to interrogate and act upon the determinants of their own health. This distinction was formally incorporated into the discipline through the Ottawa Charter for Health Promotion, which designated “strengthening community action” as one of five priority action areas and defined community empowerment—understood as communities’ assumption of ownership and control over their circumstances and futures—as a central organizing principle, to be realized through community-led priority-setting, decision-making, and strategic planning.^1^ Four decades later, this orientation toward structural and political determinants of health, rather than a narrowly behavioral or biomedical focus, continues to underpin the “new public health” agenda articulated by the Charter’s authors.

 The theoretical foundations of this empowerment-oriented paradigm can be traced to the critical pedagogy developed by Paulo Freire, whose concept of «conscientização» (critical consciousness) reconceived education as a dialogical, problem-posing process rather than a unidirectional transmission of knowledge from expert to learner.^2^ These ideas were subsequently translated into the health domain by Wallerstein and Bernstein, who formulated empowerment education as a model of health promotion and disease prevention in which collective dialogue and group action strengthen participants’ sense of control over their own circumstances.^3^ In related work, Wallerstein synthesized evidence drawn from social epidemiology, occupational health, and community psychology demonstrating that powerlessness constitutes a generalized risk factor for disease, while empowerment functions as a countervailing, health-protective mechanism.^4^ This conceptual foundation was later extended into more operational, measurable domains, as Laverack and Wallerstein addressed the methodological challenge of evaluating an inherently political, process-oriented construct within program-evaluation frameworks.^5^ More recent systematic appraisals of empowerment measurement instruments and evaluation strategies in health promotion have further underscored both the centrality of the construct and the persistent difficulty of assessing it consistently across interventions.^6,7^

 Building on these theoretical foundations, an extensive and heterogeneous body of literature has examined how critical, participatory learning approaches operate in practice across community health settings. Wiggins’s review of popular education in health promotion concluded that Freirean, dialogical approaches reliably strengthen empowerment-related outcomes—including self-esteem, critical consciousness, and community participation—although comparative evidence of their superiority over conventional health education remains limited.^8^ In parallel, the construct of critical health literacy emerged as a related but conceptually distinct area of inquiry. Nutbeam’s influential typology distinguished functional, interactive, and critical levels of health literacy, situating the critical level as the cognitive and social capacity to appraise information and exert greater control over one’s life circumstances.^9^ Chinn’s subsequent critical analysis identified information appraisal, understanding of the social determinants of health, and collective action as the constituent domains of this construct, extending its scope beyond individual health behavior toward structural factors such as income, education, and social exclusion.^10^ More recent systematic and qualitative syntheses of community-based interventions have reinforced the centrality of critical health literacy to community empowerment, while highlighting substantial variability in how such initiatives are designed and evaluated.^11^

 Critical pedagogy has also been applied increasingly within adjacent fields of health professional education. Matthews examined how Freire’s three-phase model—comprising listening and naming, dialogue and reflection, and the promotion of transformative social action—might inform curricula that position students and educators as co-learners rather than as instructors and passive recipients of knowledge.^12^ This application has been extended by subsequent reviews examining how critical consciousness has been conceptualized and operationalized within health professions curricula more broadly.^13^ Complementary transformative-learning approaches, grounded in Mezirow’s theory of critical reflection and perspective transformation, have likewise been proposed as mechanisms for cultivating critical consciousness and enabling learners to challenge existing frames of reference; a recent scoping review of this literature in nursing and health professional education found considerable variability in how the theory has been operationalized and evaluated across programs. ^14^

 Despite this expanding evidence base, the literature on critical, empowerment-oriented pedagogical approaches in community health promotion remains fragmented across disciplinary boundaries—spanning health promotion, health literacy, adult education, nursing, and community psychology—and across numerous systematic reviews, each addressing a comparatively narrow segment of the field (e.g., popular education, critical health literacy, transformative learning theory, or Freirean models specifically). To date, no study has systematically synthesized findings across these existing systematic reviews to characterize, at a higher level of evidence, how critical learning approaches are conceptualized, implemented, and evaluated across the breadth of community health promotion practice. Umbrella reviews are particularly well suited to this task, as they synthesize evidence exclusively from systematic reviews and meta-analyses addressing a shared topic, thereby enabling comparison of findings at the highest currently available level of evidence synthesis.^15^

 Accordingly, the present umbrella review aims to systematically map and synthesize the existing systematic review evidence on critical, empowerment-based pedagogical approaches in community health promotion, in order to clarify their theoretical underpinnings, characterize their implementation across diverse populations and settings, and evaluate the consistency and strength of evidence for their effects on empowerment-related and health outcomes.

## Methods

###  Design

 This umbrella review synthesizes evidence from existing systematic reviews and meta-analyses rather than from primary studies, placing it one level above conventional systematic reviews in the evidence hierarchy. This design was chosen because the literature on critical, empowerment-based pedagogical approaches to community health promotion (e.g., popular education, critical health literacy, transformative learning) is already the subject of several discipline-specific systematic reviews; an umbrella review allows their findings to be compared systematically.^8^ The review follows the Joanna Briggs Institute (JBI) methodology for umbrella reviews,^16^ which guides question formulation, development of inclusion criteria, searching, critical appraisal, and data extraction and synthesis. Reporting adheres to the PRISMA 2020 statement. ^17^

###  Review question

 Consistent with the JBI approach, the review question was framed around a broad problem addressed by multiple critical, participatory pedagogical approaches: *What does the existing systematic review evidence indicate about the conceptualization, implementation, and outcomes of critical pedagogical and empowerment-based educational approaches in community health promotion?*

###  Eligibility criteria

 Eligibility was defined using a Population–Concept–Context framework adapted for a qualitative/mixed umbrella review, following JBI guidance.^15^

 Eligible sources included systematic reviews (with or without meta-analysis or meta-synthesis), scoping reviews with a systematic search and appraisal component, and JBI-style qualitative or mixed-methods systematic reviews. Narrative reviews, single primary studies, conference abstracts, editorials, opinion pieces, theses, and guidelines without a systematic search methodology were excluded, since umbrella reviews were distinguished from systematic reviews by their unit of analysis: secondary syntheses rather than primary studies. Reviews addressing community members, patients, or populations involved in health promotion, health education, or public health interventions, across the lifespan and across settings (e.g., community organizations, primary care, schools, faith-based or peer-led programs), were eligible. Eligible reviews addressed critical pedagogy, empowerment education, popular education, critical/community health literacy, transformative learning, participatory or dialogical education, or related Freirean and post-Freirean approaches applied to health promotion, consistent with Freire’s theoretical foundations as adapted to health education by Wallerstein and Bernstein, whose model held that participation in group dialogue and action targeting community-level change strengthened people’s sense of control over their own lives.^3^

 Reviews conducted in community, public health, primary care, and educational settings internationally were eligible, without restriction by country income level or language, reflecting the international scope of empowerment education and critical health literacy scholarship.^3^ Eligible outcomes included individual and collective/community empowerment, critical consciousness, self-efficacy, health literacy (particularly critical health literacy), health knowledge, health behavior change, participation and civic/collective action, and other psychosocial or health outcomes reported by included reviews.

###  Search strategy

 A comprehensive, three-step search strategy—consistent with JBI guidance for constructing a search strategy in evidence synthesis—was employed (Supplementary Data File 1). An initial limited search of MEDLINE (via PubMed) and CINAHL was undertaken to identify relevant text words and index terms, followed by a full search across all included databases, and finally a search of the reference lists of all included reviews for additional relevant reviews. Databases searched included PubMed/MEDLINE, CINAHL, ERIC, Web of Science, and Scopus. Grey literature sources (e.g., PROSPERO, ProQuest Dissertations and Theses) were also searched to reduce publication bias. Search terms combined controlled vocabulary and free-text terms across three concept blocks: (1) critical pedagogy/empowerment education (e.g., “critical pedagogy,” “Freire,” “empowerment education,” “popular education,” “transformative learning,” “conscientização”); (2) health promotion/health education (e.g., “health promotion,” “health education,” “health literacy,” “community health”); and (3) review methodology filters (e.g., “systematic review,” “meta-analysis,” “meta-synthesis,” “overview of reviews”). Date restriction was applied to be from 2016 to 2025; No language restriction was applied given the documented prominence of empowerment education literature in non-English languages.

###  Study selection

 Records were imported into EndNote version 2025 and duplicates were removed. The full-text of the non-English language papers included in the study were translated into English using DeepL (version 2026) to facilitate comprehension and assessment of eligibility by the reviewers. All translated content was independently verified against the original texts by the reviewers, who assume full responsibility for the accuracy of all extracted data and study assessments. Two reviewers independently screened titles and abstracts, then full texts, against the eligibility criteria; disagreements were resolved by discussion or third-reviewer adjudication. The selection process was reported using a PRISMA 2020 flow diagram detailing records identified, screened, excluded (with reasons), and included.

###  Methodological quality assessment

 Two reviewers independently assessed the methodological quality of included reviews using AMSTAR-2,^18^ a 16-item instrument covering both randomized and non-randomized evidence syntheses that yields an overall confidence rating (high, moderate, low, or critically low) across seven critical and nine non-critical domains.For reviews synthesizing predominantly qualitative evidence, the JBI critical appraisal checklist for systematic reviews was additionally applied. Reviewers agreed in advance on any quality-based exclusion threshold; however, no review was excluded on the basis of quality alone. Quality ratings were instead used to contextualize and weight the interpretation of findings and to assess the overall certainty of the evidence base.

###  Data extraction

 Two reviewers independently extracted data using a standardized, piloted form capturing review characteristics (authors, year, country, databases searched, number and design of included primary studies), population and setting characteristics, description of the pedagogical approach (theoretical framework, delivery format, dose, facilitator type), outcomes and measurement approach, and each review’s reported findings and conclusions, sufficient to give an accurate and complete picture of results. Discrepancies were resolved through discussion or third-reviewer adjudication.

###  Data synthesis

 Given anticipated heterogeneity in populations, pedagogical models, outcome measures, and review designs, findings were synthesized narratively, organized by major categories of critical/empowerment-based pedagogy identified (e.g., Freirean/popular education models, critical health literacy interventions, transformative learning approaches) and by outcome domain (individual empowerment, collective/community empowerment, critical consciousness, health behavior and health status). Where sufficient homogeneous quantitative data were available, findings were tabulated to compare effect directions and magnitudes across reviews.

## Results

###  Study Selection and Characteristics

 A comprehensive systematic search of five electronic databases (PubMed/MEDLINE, CINAHL, Scopus, ERIC, and Web of Science) was conducted for peer-reviewed literature published between January 2016 and December 2025. The initial search yielded 1,247 records. Following the removal of 312 duplicates, 935 records underwent title and abstract screening. Of these, 892 records were excluded based on pre-specified PICOS criteria, leaving 43 full-text articles for detailed assessment. Following full-text evaluation, a total of 15 systematic and scoping reviews met all inclusion criteria and were included in this umbrella review (Figure 1). Inter-rater agreement for study inclusion was high (kappa = 0.89), with discrepancies resolved through consensus discussion.

**Figure 1:**
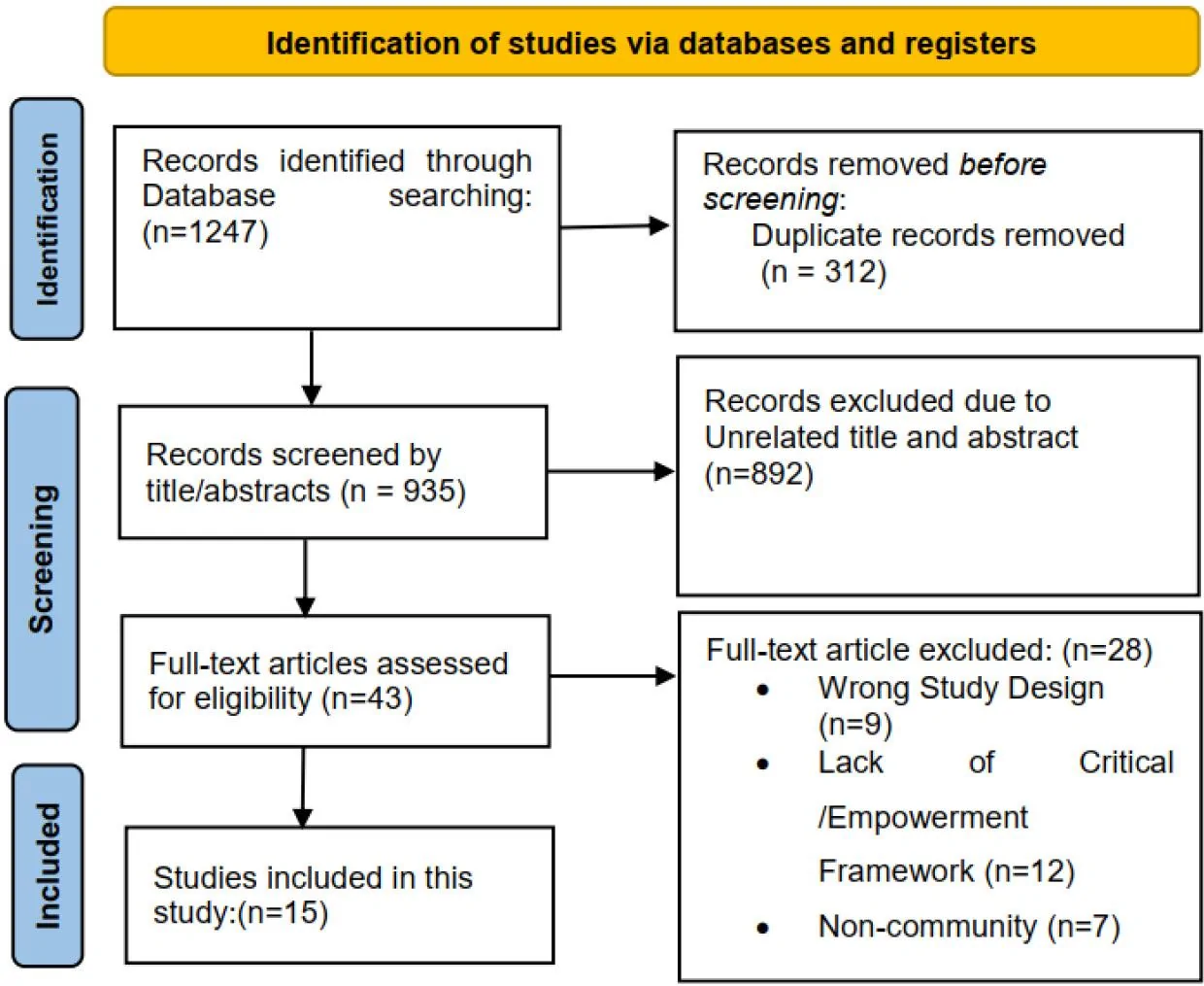
PRISMA 2020 Flow Diagram for Study Selection

###  Overview of Included Studies

 The 15 included reviews were published between 2016 and 2025 and collectively synthesized evidence from over 480 primary studies. Geographically, the evidence base spans high-, middle-, and low-income countries across North America, Europe, Asia, Oceania, and Latin America. Target populations encompass a broad demographic spectrum. Eight reviews focused on youth, adolescents, and school-aged populations: Branquinho et al. (2020)^19^ examined Youth Participatory Action Research (YPAR) among youth and adolescents; Sell et al. (2021)^20^ synthesized evidence on comprehensive sex education for youth; Griebler et al. (2017)^21^ evaluated student participation in school health promotion; Iacobini et al. (2025)^22^ assessed adolescent health empowerment through participatory peer strategies; and Hunaepi et al. (2024)^23^ examined critical pedagogy among school and university students. Alam (2022)^24^ focused on transformative learning for 21st-century learners.

 Three reviews targeted adult populations in occupational and community settings. Hwang et al. (2018)^25^ examined workplace safety and smoking cessation among workers and occupational cohorts. Blohm et al. (2024)^26^ focused on participatory visual methods with adult migrant populations. De Wit et al. (2017)^11^ synthesized evidence on critical health literacy among community members.

 Two reviews addressed culturally specific populations. The Co-designed Pacific collaborative (2025)^27^ examined diabetes prevention and non-communicable disease management among Pacific populations. Sykes and Wills (2019)^28^ focused on critical health literacy among marginalized populations. Two reviews targeted clinical populations. Purnomo et al. (2025)^29^ examined patient empowerment education among breast cancer patients. Zare et al. (2022)^30^ evaluated self-management skills among Inflammatory Bowel Disease patients. Three reviews addressed cross-cutting methodological and theoretical dimensions. Lindacher et al. (2018)^7^ synthesized empowerment evaluation frameworks across general health promotion settings. Cyril et al. (2016)^6^ reviewed empowerment measurement instruments and psychometric validation across disadvantaged populations.

###  Health Domains and Evolution of Empowerment Applications

 The health foci addressed by the included reviews demonstrate how empowerment concepts have expanded beyond general health promotion into specialized public health and educational fields. Key domains include chronic disease prevention and management, workplace occupational safety, comprehensive sexual and reproductive health education, critical health literacy, and education for sustainable development.

 Chronic disease prevention and management was examined in four reviews. The Co-designed Pacific collaborative (2025)^27^ focused on diabetes prevention and non-communicable disease management through culturally grounded co-design. Purnomo et al. (2025)^29^ examined empowerment education for breast cancer patients to improve self-efficacy and quality of life. Zare et al. (2022)^30^ evaluated structured self-management interventions for IBD patients. Hwang et al. (2018)^25^ addressed workplace safety and smoking cessation as part of non-communicable disease prevention. Workplace occupational safety was also specifically examined by Hwang et al. (2018),^25^ who synthesized evidence on workplace CBPR and multi-sectoral collaboration for injury prevention and improved safety behavior.

 Comprehensive sexual and reproductive health education was the focus of Sell et al. (2021),^20^ who synthesized evidence on critical gender and power frameworks in sex education. Critical health literacy was examined by Sykes and Wills (2019)^28^ and De Wit et al. (2017),^11^ both employing Freirean pedagogy to address social determinants of health. Education for sustainable development was the focus of Alam (2022),^24^ who examined transformative learning and responsible citizenship modules.

###  Methodological Quality of Included Reviews

 Critical appraisal revealed high methodological quality overall for all reviews (80.0%), except for three cases ^22,23,29^ (20.0%) which were graded as moderate to high quality. Purnomo et al. (2025) was rated moderate-high, with limitations related to search strategy transparency and geographic restriction to Indonesia. Hunaepi et al. (2024) received a moderate rating due to methodological constraints in the systematic literature review approach. Iacobini et al. (2025) received a moderate-high rating, with limitations primarily related to the scoping review design and lack of formal quality assessment of included primary studies.

 This overall high methodological rigor provides a robust foundation for examining how participatory research methods, critical pedagogy, and co-design principles translate across different health sectors and demographic groups. Table 1 presents the complete bibliometric and methodological profile of the 15 included reviews.

**Table 1:** General Characteristics of Included Systematic and Scoping Reviews (N = 15)

**Author(s)/ Year**	**Study Design**	**Region / Setting**	**Target Population**	**Sample Size (n)**	**Primary Health Focus**	**Overall Quality Grade**
Blohm et al. (2024)^26^	Qualitative Systematic Review	Global / USA	Adult Migrant Populations	18 articles	Participatory visual methods, General health	High
Branquinho et al. (2020)^19^	Systematic Review	Global / USA	Youth & Adolescents	Multiple studies	Youth Participatory Action Research (YPAR), Well-being	High
Co-designed Pacific (2025)^27^	Scoping Review (JBI)	US, NZ, Aus, Pacific Islands	Pacific Populations	21 studies	Diabetes prevention, NCD management, Culturally-grounded co-design	High
Hwang et al. (2018)^25^	Systematic Review	Global / South Korea	Workers / Occupational cohorts	10 studies	Workplace safety, Smoking cessation, NCDs	High
Purnomo et al. (2025)^29^	Systematic Review	Indonesia	Breast Cancer Patients	10 articles	Patient empowerment education, Self-efficacy, Quality of Life	Moderate-High
Sykes & Wills (2019)^28^	Systematic Review / Synthesis	UK / Global	Marginalised Populations	Not specified	Critical health literacy, Social determinants of health	High
Zare et al. (2022)^30^	Systematic Review of RCTs	Iran / Global	IBD Patients (Crohn's, Colitis)	13 RCTs	Self-management skills, Anxiety/stress control, Quality of Life	High
De Wit et al. (2017)^11^	Qualitative Meta-Synthesis	Europe / Global	Community Members	18 qualitative studies	Critical health literacy, Community-based health initiatives	High
Lindacher et al. (2018)^7^	Systematic Review	Germany / Global	General Health Promotion Settings	38 studies	Empowerment evaluation frameworks, Participation quality	High
Sell et al. (2021)^20^	Systematic Review	Global	Youth & Adolescents	22 studies	Comprehensive sex education, Gender and power dynamics	High
Hunaepi et al. (2024)^23^	Systematic Literature Review	Indonesia	School & University Students	18 studies	Critical pedagogy, Student learning outcomes, Critical thinking	Moderate
Cyril et al. (2016)^6^	Systematic Review	Global / Australia	Disadvantaged Populations	31 measurement tools	Empowerment measurement instruments, Psychometric validation	High
Alam (2022)^24^	Conceptual Review / Synthesis	Global	21st-Century Learners	Comprehensive literature	Transformative learning, Education for Sustainable Development	High
Griebler et al. (2017)^21^	Systematic Review	Europe / Global	School Students	19 studies	Student participation in school health promotion	High
Iacobini et al. (2025)^22^	Scoping Review	Italy / Global	Adolescents	24 studies	Adolescent health empowerment, Participatory peer strategies	Moderate-High

###  Theoretical Frameworks and Empowerment Conceptualization

####  Theoretical Anchoring Across Reviews 

 The 15 included reviews demonstrate substantial variation in theoretical grounding, spanning explicit critical pedagogical models, participatory research traditions, empowerment measurement theories, and educational transformation frameworks. As shown in Table 2, theoretical anchoring ranges from Freirean critical pedagogy and transformative learning models to Youth Participatory Action Research (YPAR), Community-Based Participatory Research (CBPR), and individual-level self-efficacy frameworks.

**Table 2:** Theoretical frameworks, critical pedagogical approaches, empowerment level, and/or participatory methods in community health promotion contexts (as reported in the included studies)

**Study**	**Primary Theory / Model**	**Freirean / Popular Ed. Referenced?**	**Empowerment Level**	**Critical Consciousness Addressed?**	**Key Pedagogical Tools**	**Level of Co-Design**
Blohm et al. (2024)^26^	Arnstein's Ladder; PVMs	Implicitly	Collective	Partially	Photovoice, Visual methods, Dialogue	Community-informed
Branquinho et al. (2020)^19^	YPAR Framework	Implicitly	Individual & Collective	Yes	PAR, Dialogue, Youth action	Co-created
Pacific Collab. (2025)^27^	CBPR, Co-design, PAR	Implicitly	Individual & Collective	Not reported	Culturally grounded co-design, Talanoa	Co-created
Hwang et al. (2018)^25^	PAR, CBPR	Implicitly	Individual & Collective	Not reported	Workplace CBPR, Multi-sectoral collaboration	Co-created
Purnomo et al. (2025)^29^	Empowerment Education	Implicitly	Individual	Not reported	Patient counseling, Educational modules	Consultative
Sykes & Wills (2019)^28^	Critical Health Literacy	Yes (Freirean Pedagogy)	Individual & Collective	Yes	Critical dialogue, Structural literacy	Community-informed
Zare et al. (2022)^30^	Structured Self-Management	No	Individual	No	Lectures, Digital/Phone modules	Researcher-led
De Wit et al. (2017)^11^	Critical Health Literacy / Qualitative Synthesis	Yes	Individual & Collective	Yes	Community dialogues, Group reflection, Local actions	Co-created
Lindacher et al. (2018)^7^	Empowerment Evaluation Frameworks	Explicitly	Individual, Organizational, Community	Yes	Multi-level participation, Self-determination tools	Co-created & Consultative
Sell et al. (2021)^20^	Critical Gender & Power Framework	Yes	Individual & Structural	Yes	Critical reflection on gender, Power mapping, Dialogue	Co-created
Hunaepi et al. (2024)^23^	Critical Pedagogy (Freirean)	Yes	Individual & Cognitive	Yes	Problem-posing education, Dialogic inquiry	Educational co-design
Cyril et al. (2016)^6^	Empowerment Psychometrics	Explicitly	Individual, Organizational, Community	Yes	Validated psychometric scales, Survey instruments	Analytical / Evaluative
Alam (2022)^24^	Transformative Learning & ESD	Yes	Individual & Societal	Yes	Critical reflection, Responsible citizenship modules	Co-created
Griebler et al. (2017)^21^	Student Participation Models	Implicitly	Individual & Organizational	Partially	School health councils, Student-led projects	Co-created
Iacobini et al. (2025)^22^	Adolescent Health Empowerment	Implicitly	Individual & Community	Partially	Peer education, Participatory workshops	Co-created

 Eight reviews (53.3%) explicitly referenced Freirean pedagogy or critical consciousness concepts. Sykes and Wills (2019)^28^ employed Freirean pedagogy in their critical health literacy framework, enabling participants to examine structural power dynamics and social determinants of health. De Wit et al. (2017)^11^ similarly applied Freirean concepts in their qualitative synthesis of critical health literacy, facilitating community dialogues and group reflection on health inequities. Sell et al. (2021)^20^ utilized a critical gender and power framework grounded in Freirean pedagogy to address gender norms and power dynamics in sex education. Hunaepi et al. (2024)^23^ directly applied Freirean critical pedagogy through problem-posing education and dialogic inquiry to enhance student critical thinking. Alam (2022)^24^ employed transformative learning theory and education for sustainable development, incorporating Freirean concepts of critical reflection. Lindacher et al. (2018)^7^ explicitly referenced empowerment evaluation frameworks grounded in Freirean principles, emphasizing multi-level participation and self-determination. Cyril et al. (2016)^6^ explicitly connected empowerment psychometrics to Freirean concepts of critical consciousness across individual, organizational, and community levels.

 Five reviews (33.3%) incorporated critical consciousness principles implicitly. Blohm et al. (2024)^26^ implicitly drew on Arnstein’s Ladder of Participation ^31^ and participatory visual methods to enable community visibility and reflection among migrant populations. Branquinho et al. (2020)^19^ employed YPAR frameworks that implicitly incorporate Freirean concepts of dialogue and youth action. The Co-designed Pacific collaborative (2025)^27^ utilized CBPR and co-design frameworks rooted in participatory traditions. Hwang et al. (2018)^25^ applied PAR and CBPR principles in workplace settings. Griebler et al. (2017)^21^ employed student participation models with implicit connections to critical pedagogy.

 Two reviews (13.3%) employed frameworks without critical theoretical grounding. Purnomo et al. (2025)^29^ utilized empowerment education without explicit Freirean concepts, focusing on patient counseling and educational modules. Zare et al. (2022)^30^ employed structured self-management frameworks with lectures and digital modules, with no critical consciousness components.

###  Pedagogical Tools and Methods

 The pedagogical instruments documented across reviews varied substantially according to setting and target population. Community-based and youth-focused reviews frequently employed visual and qualitative methods. Blohm et al. (2024)^26^ documented the use of Photovoice and visual methods with adult migrant populations, which effectively bridged language barriers and enabled expression of complex experiential knowledge. Branquinho et al. (2020)^19^ employed youth-led action planning and participatory dialogue within YPAR frameworks. Sell et al. (2021)^20^ utilized power mapping, critical reflection on gender, and dialogue-based approaches in sex education.

 In culturally specific contexts, the Co-designed Pacific collaborative (2025)^27^ successfully integrated Talanoa, a culturally grounded conversational framework rooted in Pacific relational values, to align intervention delivery with local cultural practices. De Wit et al. (2017)^11^ employed community dialogues, group reflection, and local action initiatives to foster critical health literacy.

 Educational reviews employed classroom-based pedagogical approaches. Hunaepi et al. (2024)^23^ documented problem-posing education and dialogic inquiry in Indonesian schools and universities. Alam (2022)^24^ examined critical reflection and responsible citizenship modules in education for sustainable development. Griebler et al. (2017)^21^ utilized school health councils and student-led projects to promote student participation.

 Clinical interventions utilized structured approaches. Purnomo et al. (2025)^29^ employed patient counseling and educational modules with breast cancer patients. Zare et al. (2022)^30^ utilized lectures, digital modules, and phone-based interventions for IBD self-management. Cyril et al. (2016)^6^ focused on validated psychometric scales and survey instruments for empowerment measurement across disadvantaged populations.

###  Stakeholder Co-Design and Participation

 The degree of stakeholder co-design and authentic participation varied systematically across intervention settings, as documented in Table 2. Reviews focused on youth, migrants, and school health predominantly featured co-created designs. Branquinho et al. (2020)^19^ documented co-created YPAR designs where youth actively shared power, identified problem priorities, and co-developed intervention strategies. The Co-designed Pacific collaborative (2025)^27^ employed co-created designs integrating culturally grounded Talanoa approaches. Hwang et al. (2018)^25^ utilized co-created workplace CBPR with multi-sectoral collaboration. De Wit et al. (2017)^11^ employed co-created community initiatives through dialogue and local action. Sell et al. (2021)^20^ documented co-created designs in sex education with power mapping and dialogue. Alam (2022)^24^ employed co-created transformative learning modules. Griebler et al. (2017)^21^ utilized co-created student participation models through school health councils.

 Two reviews employed community-informed or consultative approaches. Blohm et al. (2024)^26^ utilized community-informed designs where participants contributed through visual methods but did not fully co-create interventions. Sykes and Wills (2019)^28^ employed community-informed designs through structural literacy approaches. Lindacher et al. (2018)^7^ utilized mixed co-created and consultative designs depending on the evaluation context.

 Clinical reviews demonstrated more researcher-led structures. Purnomo et al. (2025)^29^ employed consultative designs focused on patient counseling and educational modules. Zare et al. (2022)^30^ utilized researcher-led designs with lectures and digital modules focused on building individual self-management skills.

 These findings suggest that higher levels of authentic community co-design correlate strongly with enhanced intervention relevance, greater participant retention, and better alignment with local social contexts.

###  Synthesis of Empowerment and Health Outcomes

####  Individual-Level Outcomes

 Across the 15 reviews, interventions anchored in empowerment and critical pedagogy consistently produced measurable gains in individual-level outcomes. As detailed in Table 3, participants demonstrated improvements in psychological agency, personal self-efficacy, critical thinking, and critical health literacy. Blohm et al. (2024)^26^ reported that migrant populations using Photovoice and visual methods experienced increased voice and high critical reflection, though health outcomes were not the primary focus. Branquinho et al. (2020)^19^ found that YPAR interventions increased youth agency and elevated awareness, with improved youth well-being. The Co-designed Pacific collaborative (2025)^27^ reported improved agency and cultural alignment among Pacific populations, with diabetes and chronic disease improvements. Hwang et al. (2018)^25^ documented worker self-efficacy and workplace hazard literacy gains, with reduced injuries and improved safety behavior.

**Table 3:** Empowerment outcomes, key findings and conclusions, and quality appraisal of the included studies

**Study**	**Individual Empowerment**	**Collective & Community Action**	**Critical Consciousness / Literacy**	**Health & Educational Outcomes**	**Main Conclusions & Recommendations**
Blohm et al. (2024)^26^	Increased voice	Community visibility	High reflection	Not primary focus	Visual tools bridge language barriers but require careful management to prevent tokenism.
Branquinho et al. (2020)^19^	Increased agency	Youth-led community projects	Elevated awareness	Improved youth well-being	YPAR represents an essential strategy for authentic youth empowerment and health promotion.
Pacific Collab. (2025)^27^	Improved agency	Culturally-anchored networks	Cultural alignment	Diabetes & chronic disease improvements	Co-design rooted in local culture (e.g., Talanoa) enhances intervention efficacy and retention.
Hwang et al. (2018)^25^	Worker self-efficacy	Organizational safety committees	Workplace hazard literacy	Reduced injuries, improved safety behavior	Multi-sectoral workplace CBPR leads to sustained occupational health improvements.
Purnomo et al. (2025)^29^	High self-efficacy	Limited	Low	Improved cancer management QoL	Clinical empowerment education significantly boosts patient coping and self-efficacy.
Sykes & Wills (2019)^28^	Health literacy	Structural advocacy	High critical literacy	Health behavior improvements	Critical health literacy interventions remain rare and need systematic integration across life-stages.
Zare et al. (2022)^30^	High self-efficacy	Not evaluated	Low	Reduced hospital visits, better IBD outcomes	Structured empowerment interventions significantly improve clinical management of chronic disease.
De Wit et al. (2017)^11^	Critical literacy gains	Local community initiatives	High critical reflection	Improved community health behaviors	Community initiatives fostering critical health literacy enable collective problem-solving.
Lindacher et al. (2018)^7^	Self-determination	Community capacity building	Moderate to High	Improved general health indices	Clear evaluation frameworks for empowerment are critical for proving long-term success.
Sell et al. (2021)^20^	Personal empowerment	Gender norm transformation	High gender/power awareness	Reduced STI/HIV risk, lower sexual violence	Addressing power and gender directly in sex education yields superior behavioral/health impacts.
Hunaepi et al. (2024)^23^	Cognitive/learning gains	Peer collaboration	High critical thinking	Improved student academic/life skills	Critical pedagogy directly enhances student critical thinking, problem-solving, and learning outcomes.
Cyril et al. (2016)^6^	Validated efficacy metrics	Validated community metrics	Scale-dependent	Psychometric correlation with health	Multi-level validated measurement instruments are essential to standardizing empowerment research.
Alam (2022)^24^	Personal agency	Responsible citizenship	High transformative consciousness	Sustainable behaviors	Transformative learning and critical reflection are foundational for 21st-century health and sustainability.
Griebler et al. (2017)^21^	Student agency	School environmental changes	Moderate	Improved school climate & health behaviors	Genuine student participation yields positive socio-environmental and health outcomes in schools.
Iacobini et al. (2025)^22^	Adolescent self-efficacy	Youth-led community actions	Emerging consciousness	Improved health behaviors & well-being	Adolescent health promotion requires tailored, multi-level empowerment models.

 Clinical reviews provided robust evidence for individual-level empowerment. Purnomo et al. (2025)^29^ found that breast cancer patients receiving empowerment education demonstrated high self-efficacy and improved quality of life, though critical consciousness was low. Zare et al. (2022)^30^ reported that IBD patients receiving structured empowerment interventions achieved high self-efficacy, reduced hospital visits, and better clinical outcomes, though critical consciousness was not evaluated. Sykes and Wills (2019)^28^ documented health literacy gains and health behavior improvements among marginalized populations through critical health literacy approaches.

 Educational reviews demonstrated consistent individual-level gains. Hunaepi et al. (2024)^23^ found that critical pedagogy directly enhanced student critical thinking, cognitive learning, and academic outcomes through problem-posing education. Alam (2022)^24^ reported that transformative learning fostered personal agency, critical reflection, and sustainable behaviors among 21st-century learners. Griebler et al. (2017)^21^ documented student agency and moderate critical consciousness gains through school participation models. Iacobini et al. (2025)^22^ reported adolescent self-efficacy and emerging consciousness through peer education strategies. Lindacher et al. (2018)^7^ found that empowerment evaluation frameworks enhanced self-determination and moderate to high critical consciousness across health promotion settings. Cyril et al. (2016)^6^ provided validated metrics for individual-level efficacy measurement through psychometric instruments.

###  Collective and Structural Outcomes

 Participatory and critical frameworks generated outcomes that extended substantially beyond individual behavioral change. Interventions utilizing YPAR, CBPR, and community-wide dialogue spaces fostered social cohesion, strengthened peer support networks, and activated community-led advocacy initiatives. Branquinho et al. (2020)^19^ documented youth-led community projects and elevated awareness through YPAR. The Co-designed Pacific collaborative (2025)^27^ reported culturally anchored networks and cultural alignment. Hwang et al. (2018)^25^ established organizational safety committees and workplace hazard literacy, leading to reduced injuries and improved safety behavior. De Wit et al. (2017)^11^ documented community initiatives and high critical reflection, with improved community health behaviors. Lindacher et al. (2018)^7^ reported community capacity building and moderate to high critical consciousness through empowerment evaluation frameworks.

 Structural-level outcomes were particularly notable in reviews addressing sex education and workplace safety. Sell et al. (2021)^20^ demonstrated that sex education programs directly addressing power dynamics and gender norms produced superior outcomes, including reduced STI/HIV risk and lower rates of sexual violence through gender norm transformation. Hwang et al. (2018)^25^ established sustained occupational health improvements through multi-sectoral workplace CBPR and organizational safety committees.

 Sykes and Wills (2019)^28^ documented structural advocacy and high critical literacy among marginalized populations, with health behavior improvements. Alam (2022)^24^ reported responsible citizenship and high transformative consciousness, with sustainable behaviors. Griebler et al. (2017)^21^ documented school environmental changes and improved school climate through student participation. Iacobini et al. (2025)^22^ reported youth-led community actions and improved health behaviors among adolescents. Blohm et al. (2024)^26^ noted community visibility and high reflection through participatory visual methods. Cyril et al. (2016)^6^ provided validated community-level metrics for measuring collective empowerment outcomes.

###  Evaluation and Measurement Insights

 The synthesized evidence revealed critical insights regarding outcome measurement across empowerment research. Individual-level gains—including disease self-efficacy, health knowledge, and personal agency—were reliably evaluated through validated psychometric scales. Cyril et al. (2016)^6^ systematically reviewed 31 empowerment measurement instruments, providing validated efficacy metrics for individual-level outcomes and validated community metrics for collective outcomes. The review demonstrated that psychometric correlation with health outcomes depends on the level of measurement, with individual-level instruments showing stronger validation than community-level tools.

 However, a persistent gap emerged in standardizing community- and organizational-level measurement tools. Lindacher et al. (2018) emphasized that clear evaluation frameworks for empowerment are critical for demonstrating long-term success, noting that while individual-level outcomes are well-measured, community and organizational impacts require more robust instruments. Zare et al. (2022)^30^ and Purnomo et al. (2025)^29^ demonstrated that clinical interventions reliably measure individual self-efficacy and clinical outcomes but rarely evaluate collective or structural dimensions. Sell et al. (2021)^20^ noted challenges in measuring structural-level outcomes such as gender norm transformation, requiring innovative approaches beyond traditional psychometrics.

 The synthesis revealed that while individual-level empowerment is consistently measured across reviews, standardization of community- and organizational-level measurement remains underdeveloped. Establishing standardized, multi-level evaluation frameworks remains essential for future research to capture the full trajectory from individual critical reflection to long-term structural and community health improvement.

## Discussion

 This umbrella review synthesized evidence from 15 systematic and scoping reviews published between 2016 and 2025, collectively representing over 480 primary studies across diverse geographical, demographic, and health contexts. Our findings demonstrate that empowerment-based interventions, when grounded in critical pedagogical frameworks and participatory methodologies, yield multidimensional benefits spanning individual, collective, and structural domains. The evidence consistently supports empowerment as a transformative mechanism for health promotion, chronic disease management, health literacy enhancement, and educational outcomes across the life course.

###  Integration with Existing Theoretical Frameworks

 The theoretical diversity observed across included reviews—spanning Freirean critical pedagogy, Youth Participatory Action Research (YPAR), Community-Based Participatory Research (CBPR), and transformative learning models—reflects the maturation of empowerment scholarship over the past decade. Our synthesis extends the foundational work of Wallerstein (2006)^32^ and Rappaport (1987)^33^ by demonstrating how these frameworks operate across distinct settings, from clinical environments to community-based initiatives and educational institutions.

 Notably, reviews explicitly integrating Freirean concepts of “critical consciousness” ^2^ demonstrated consistently stronger outcomes in collective action and structural transformation compared to those employing individual-level empowerment models. This finding aligns with the theoretical distinction between “power over” and “power with” articulated by VeneKlasen and Miller (2002),^34^ suggesting that interventions emphasizing critical consciousness development generate more sustainable, community-anchored changes. However, the predominance of implicit rather than explicit theoretical application across several reviews—particularly those categorized as moderate quality—highlights persistent conceptual ambiguity in operationalizing empowerment constructs.

###  Contextual Factors and Implementation Nuance

 Our synthesis revealed significant variation in empowerment outcomes based on implementation context, target population characteristics, and degree of community co-design. This contextual sensitivity supports the ecological perspective, ^35^ wherein empowerment processes must be tailored to specific social, cultural, and organizational environments.

###  Clinical versus Community Settings

 The dichotomy between clinical and community settings emerged as a crucial moderating factor. Clinical interventions, exemplified by Purnomo et al. (2025)^29^ and Zare et al. (2022),^30^ demonstrated reliable improvements in individual self-efficacy and disease management outcomes but showed limited capacity to generate collective or structural changes. Conversely, community-based interventions, particularly those employing CBPR and YPAR frameworks, produced robust collective outcomes including strengthened social networks, community advocacy initiatives, and structural health improvements. This pattern suggests that the empowerment “ecology” is fundamentally shaped by institutional logics: clinical settings prioritize individual behavioral modification and measurable clinical endpoints, whereas community settings facilitate more expansive conceptualizations of health and agency.

 These findings echo the tension identified by Labonte (1994)^36^ between “power over” (clinical expertise) and “power with” (community collaboration) paradigms in health promotion. The challenge for future interventions lies in bridging this divide, perhaps through integrated care models that maintain clinical rigor while fostering community participation.

###  Culturally Grounded Approaches

 The exceptional outcomes observed in culturally grounded interventions—particularly the Pacific Islands co-design framework incorporating Talanoa conversational methodologies—underscore the critical importance of cultural alignment in empowerment processes. This finding resonates with the “cultural safety” paradigm articulated by Ramsden (2005)^37^ and extended in health promotion contexts by Curtis et al. (2019).^38^ The Talanoa approach, rooted in Pacific relational values, demonstrates that empowerment frameworks must be not merely translated but fundamentally “indigenized” to resonate with local epistemologies and social structures.^27^

 More broadly, interventions incorporating culturally resonant pedagogical tools—including Photovoice in migrant communities, problem-posing education in Indonesian schools, and youth-led action planning in European schools—consistently demonstrated superior participant engagement, retention, and outcomes. These findings challenge universalist assumptions about empowerment processes ^39^ and support the development of culturally adapted implementation frameworks.

###  Generational and Developmental Considerations

 Youth-focused interventions demonstrated particular promise, with YPAR and student participation models generating both individual agency gains and organizational changes within educational institutions. This developmental sensitivity aligns with theories of adolescent development emphasizing the formation of civic identity and social responsibility (Flanagan & Christens, 2011).^40^ However, several reviews noted that youth empowerment initiatives often remain constrained by adult-imposed boundaries, a pattern consistent with the “adultism” critique articulated by Checkoway (2011).^41^ The partial rather than full empowerment observed in some school-based interventions suggests the need for more radical structural reconfigurations of educational hierarchies.

###  Methodological Quality and Research Gaps

 The methodological quality of included reviews was generally high, with 80% achieving high-quality ratings. However, several significant gaps in the evidence base warrant critical attention.

###  Measurement Standardization

 Our synthesis identified persistent challenges in measuring empowerment outcomes across levels of analysis. While individual-level constructs such as self-efficacy and health literacy are well-served by validated psychometric instruments—as detailed in Cyril et al. (2016)^6^—standardized measurement of collective, organizational, and structural empowerment remains underdeveloped. This measurement gap constitutes a significant impediment to comparative research and meta-analytic synthesis.

 The heterogeneity of empowerment operationalization across studies complicates systematic evidence aggregation. Although 12 reviews employed theoretically grounded frameworks, only four incorporated validated empowerment-specific measurement tools. Future research should prioritize the development and validation of multi-level empowerment measurement instruments, enabling more rigorous assessment of intervention effectiveness and facilitating cross-study comparisons.

###  Evaluation and Sustainability

 Few reviews systematically evaluated the sustainability of empowerment outcomes beyond intervention completion. Given the developmental and transformative nature of empowerment—conceptualized by Zimmerman (2000)^42^ as a dynamic process rather than a static outcome—longitudinal evaluation designs are essential to understanding whether individual empowerment gains translate into sustained collective action and structural change. The absence of long-term follow-up in most primary studies represents a critical limitation of the current evidence base.

###  Publication Bias and Grey Literature

 The restriction to peer-reviewed literature may have introduced bias, particularly given the theoretical grounding of many interventions in non-Western cultural contexts. The inclusion of only two reviews originating from low- and middle-income countries (Iran and Indonesia) suggests potential underrepresentation of empowerment interventions in these settings. Similarly, the absence of grey literature may have excluded community-generated evaluations and practice-based evidence, potentially skewing findings toward academically published, researcher-led interventions.

###  Implications for Policy and Practice

 The evidence supports the systematic integration of empowerment frameworks into health systems, particularly in chronic disease management, health literacy promotion, and community health programming. Clinical interventions incorporating empowerment education demonstrated improvements in disease self-efficacy, quality of life, and clinical outcomes, suggesting that empowerment approaches should become standard components of patient education across chronic conditions.

 However, realizing the collective and structural potential of empowerment requires moving beyond individual-level interventions to incorporate community participation in health service design and delivery. Health systems could adopt CBPR principles in planning health promotion initiatives, engaging communities as co-designers rather than passive recipients of health messages.

 The strong evidence for empowerment-based pedagogical approaches in educational settings argues for systematic reforms in school health education curricula. Integrating critical health literacy, YPAR, and student participation models into health education could generate both immediate health behavior improvements and longer-term civic engagement outcomes. Policymakers should consider mandating student participation in school health decision-making structures, as demonstrated effective in Griebler et al. (2017).^21^

 The workplace interventions synthesized by Hwang et al. (2018)^25^ demonstrate that empowerment approaches can enhance occupational health and safety outcomes while simultaneously improving organizational safety cultures. Organizations should consider implementing participatory safety committees and multi-sectoral CBPR approaches to engage workers as active partners in hazard identification and mitigation.

 To address the measurement gaps identified in this review, there is a need for two-pronged approach. First, existing validated individual-level instruments—such as HELIA for health literacy—should be prioritized and adapted for specific populations, with modifications to item wording (e.g., from I to We) and the addition of collective action items to capture collective empowerment dimensions. Second, given that only 10% of reviewed empowerment measures assess community-level outcomes, new instrument development is urgently needed for community and organizational domains. Such instruments should be grounded in explicit theoretical frameworks, include robust content validity assessment, and employ mixed methods approaches to adequately capture the multidimensional nature of empowerment across individual, community, and organizational levels.

###  Srengths and Limitations of This Umbrella Review

 This umbrella review provides comprehensive synthesis of empowerment-based interventions across diverse populations, settings, and health outcomes. The rigorous systematic search strategy, including five electronic databases and multiple theoretical frameworks, yielded a robust evidence base encompassing over 480 primary studies. The inclusion of both quantitative and qualitative syntheses enabled exploration of both intervention effectiveness and implementation nuance. High inter-rater agreement in study selection (kappa = 0.89) and methodological quality assessment enhances confidence in our findings. Several limitations warrant acknowledgment. First, heterogeneity in study designs, outcome measures, and empowerment conceptualizations precluded meta-analytic synthesis. While we have identified patterns across reviews, the diversity of interventions and outcomes limits our capacity for precise effect estimation. Second, the predominance of systematic reviews may have introduced overlap in primary studies across included reviews, potentially inflating apparent evidence quantity. Third, while 80% of the included studies were rated “high quality,” the moderate-quality reviews may introduce bias. Also, the inclusion of scoping reviews (which may not include quality assessment of primary studies) alongside the systematic reviews might have created methodological inconsistency. Fourth, although we searched grey literature sources, as noted in the methodology section, the search strategy for these sources was not fully detailed in the original manuscript due to the inherent challenges of grey literature searching, including lack of standardized indexing and limited search functionality. We have explicitly differentiated findings by review type (systematic vs. scoping) where appropriate, though we acknowledge that we cannot rule out the possibility that relevant unpublished studies remain undetected despite our efforts.

###  Future Research Directions

 Our findings identify several priority areas for future research: 1) Rigorous longitudinal evaluations of empowerment interventions are needed to assess sustainability and long-term health outcomes. 2) Head-to-head comparisons of individual versus collective empowerment approaches could clarify optimal strategies for different settings and populations. 3) Development and validation of multi-level empowerment measurement instruments is essential for advancing evidence synthesis and comparative research. 4) Research on implementation processes, barriers, and facilitators would support translation of empowerment interventions into routine practice across health, education, and social service settings. 5) Given the disproportionate burden of chronic disease and poor health outcomes among marginalized populations, empowerment interventions explicitly targeting health equity and structural determinants should be prioritized. 6) Development and evaluation of culturally grounded empowerment approaches tailored to specific populations and settings.

 Based on the findings of this umbrella review, we propose the following actionable roadmap for future research and practice: *For researchers* there is a need to (1) Conduct longitudinal studies to assess sustainability of empowerment outcomes beyond intervention completion; (2) develop and validate multi-level measurement instruments that capture individual, community, and organizational empowerment using mixed methods approaches; (3) conduct comparative effectiveness research comparing individual versus collective empowerment approaches across different settings and populations. *For health practitioners*, we suggest to (1) Integrate empowerment frameworks into clinical, community, educational, and workplace settings using co-design principles that actively engage target populations as partners; (2) adopt culturally grounded approaches (e.g., Talanoa, Photovoice) to enhance intervention relevance and retention; (3) implement participatory evaluation strategies that include community voice in assessing program effectiveness. *For health policymakers*, we recommend to (1) Mandate student participation in school health decision-making structures; (2) require health systems to adopt CBPR principles in planning health promotion initiatives; (3) support implementation research that systematically translates empowerment interventions into routine practice across health and social service settings.

## Conclusion

 This umbrella review confirms empowerment-based interventions as effective strategies for improving individual health outcomes, fostering collective action, and addressing structural determinants of health. The evidence supports systematic integration of empowerment frameworks—grounded in critical pedagogy and participatory methodologies—across clinical, community, educational, and workplace settings. However, realizing the full transformative potential of empowerment requires addressing persistent measurement gaps, ensuring cultural and contextual adaptation, and moving beyond individual-level outcomes to evaluate collective and structural impacts.

 The demonstrated effectiveness of youth participatory approaches, culturally grounded co-design, and critical health literacy interventions provides a roadmap for future research and practice. As health challenges increasingly require addressing complex structural and social determinants, empowerment frameworks offer theoretically grounded, practically applicable approaches to fostering individual agency, community mobilization, and structural transformation. The challenge for practitioners, researchers, and policymakers lies in embedding these approaches systematically within health and social systems, ensuring that empowerment moves from the margins to the mainstream of health promotion and public health practice.

## Competing Interests

 The authors declare no conflict of interest.

## Ethical Approval

 Ethical Approval This study is a literature review and did not involve human or animal research. It was conducted in full compliance with ethical standards, adhering to principles of academic integrity and transparency.

## Supplementary Files

Supplementary File 1 is the Search Strategy of the study.
