# Supplementary material for: Pedagogy of Empowerment; Critical Learning Approaches in Community Health Promotion: An Umbrella Review
Source: Health Promot Perspect. 2026 Aug 21;16(2):153–65. doi: 10.34172/hpp.48231 (PMC13723421; doi:10.34172/hpp.48231)
Supplement: Supplementary file 1 [file hpp-16-153-s001.pdf]

Gulnoza et al, Health Promotion Perspectives 2026;16(2):153-S1
doi:10.34172/hpp.48231
https://hpp.tbzmed.ac.ir

Supplementary Data File

Search Strategies for Umbrella Review: Pedagogy of Empowerment; Critical Learning Approaches in Community Health Promotion

Search Period: January 2016 – June 2026

1. PubMed/MEDLINE Search Strategy

Search String:

("critical pedagogy"[tiab] OR "Freire"[tiab] OR "Freirean"[tiab] OR conscientização[tiab] OR "critical consciousness"[tiab] OR "empowerment education"[tiab] OR "popular education"[tiab] OR "transformative learning"[tiab] OR "critical health literacy"[tiab] OR "participatory education"[tiab] OR "dialogical education"[tiab] OR "problem-posing education"[tiab] OR "Youth Participatory Action Research"[tiab] OR YPAR[tiab] OR "Community-Based Participatory Research"[tiab] OR CBPR[tiab])

AND

("health promotion"[MeSH] OR "health promotion"[tiab] OR "health education"[MeSH] OR "health education"[tiab] OR "public health"[MeSH] OR "public health"[tiab] OR "community health"[tiab] OR "community health promotion"[tiab] OR "health literacy"[MeSH] OR "health literacy"[tiab] OR "community health services"[MeSH])

AND

("systematic review"[Publication Type] OR "systematic review"[tiab] OR "meta-analysis"[Publication Type] OR "meta-analysis"[tiab] OR "meta-synthesis"[tiab] OR "scoping review"[tiab] OR "umbrella review"[tiab] OR "overview of reviews"[tiab] OR "review of reviews"[tiab])

2. CINAHL (Cumulative Index to Nursing and Allied Health Literature) Search Strategy

Search String:

(MH "Health Promotion+") OR (MH "Health Education+") OR (MH "Community Health+") OR (MH "Health Literacy")

AND

(MH "Critical Pedagogy") OR (MH "Empowerment") OR (MH "Transformative Learning") OR (MH "Community-Based Participatory Research+")

AND

(TI ("systematic review" OR "meta-analysis" OR "meta-synthesis" OR "scoping review" OR "umbrella review" OR "overview of reviews") OR AB ("systematic review" OR "meta-analysis" OR "meta-synthesis" OR "scoping review" OR "umbrella review" OR "overview of reviews"))

("critical pedagogy" OR Freirean OR "conscientização" OR "critical consciousness" OR "empowerment education" OR "popular education" OR "transformative learning" OR "critical health literacy" OR "participatory education" OR "dialogical education" OR "problem-posing education" OR YPAR OR CBPR)

3. Scopus Search Strategy

Search String:

TITLE-ABS-KEY

("critical pedagogy" OR Freirean OR "conscientização" OR "critical consciousness" OR "empowerment education" OR "popular education" OR "transformative learning" OR "critical health literacy" OR "participatory education" OR "dialogical education" OR "problem-posing education" OR "Youth Participatory Action Research" OR YPAR OR "Community-Based Participatory Research" OR CBPR)

AND TITLE-ABS-KEY

("health promotion" OR "health education" OR "public health" OR "community health" OR "community health promotion" OR "health literacy" OR "community health services")

AND TITLE-ABS-KEY

("systematic review" OR "meta-analysis" OR "meta-synthesis" OR "scoping review" OR "umbrella review" OR "overview of reviews" OR "review of reviews")

4. ERIC (Education Resources Information Center) Search Strategy

Search String:

(DE "Critical Pedagogy") OR (DE "Critical Thinking") OR (DE "Empowerment") OR (DE "Transformative Learning") OR (DE "Popular Education") OR (DE "Student Participation")

AND

(DE "Health Promotion") OR (DE "Health Education") OR (DE "Public Health") OR (DE "Community Health") OR (DE "Health Literacy")

AND

(TI ("systematic review" OR "meta-analysis" OR "meta-synthesis" OR "scoping review" OR "umbrella review" OR "overview of reviews") OR AB ("systematic review" OR "meta-analysis" OR "meta-synthesis" OR "scoping review" OR "umbrella review" OR "overview of reviews"))

("critical pedagogy" OR Freirean OR "conscientização" OR "critical consciousness" OR "empowerment education" OR "popular education" OR "transformative learning" OR "critical health literacy" OR "participatory education" OR "dialogical education" OR "problem-posing education" OR YPAR OR CBPR)

5. Web of Science Search Strategy

Search String:

TS=("critical pedagogy" OR Freirean OR "conscientização" OR "critical consciousness" OR "empowerment education" OR "popular education" OR "transformative learning" OR "critical health

literacy" OR "participatory education" OR "dialogical education" OR "problem-posing education" OR "Youth Participatory Action Research" OR YPAR OR "Community-Based Participatory Research" OR CBPR)

AND

TS=("health promotion" OR "health education" OR "public health" OR "community health" OR "community health promotion" OR "health literacy" OR "community health services")

AND

TS=("systematic review" OR "meta-analysis" OR "meta-synthesis" OR "scoping review" OR "umbrella review" OR "overview of reviews" OR "review of reviews")

Grey Literature and Supplementary Searches

Reference lists of all 15 included reviews were hand-searched for additional relevant reviews.
